# Simulated Video Consultations as a Learning Tool in Undergraduate Nursing: Students’ Perceptions

**DOI:** 10.3390/healthcare8030280

**Published:** 2020-08-20

**Authors:** Diana Jiménez-Rodríguez, Oscar Arrogante

**Affiliations:** 1 Department of Nursing, Physiotherapy and Medicine, University of Almeria, 04120 Almeria, Spain; 2 University Centre of Health Sciences San Rafael, San Juan de Dios Foundation, Nebrija University, 28036 Madrid, Spain; oarrogan@nebrija.es

**Keywords:** COVID-19, high fidelity simulation training, learning, nursing education, video conferencing, virtual simulation

## Abstract

Simulated video consultations, a teaching tool based on high-fidelity simulations, were implemented in response to the necessary adaptation of high-fidelity clinical simulation sessions to the online or virtual modality during the university closure due to the COVID-19 confinement. The purpose of our study was to explore the undergraduate nursing students’ satisfaction and perceptions about simulated video consultations using the high-fidelity simulation methodology. A mixed-method was utilized with 93 undergraduate nursing students using a validated satisfaction questionnaire (quantitative data), which included an observations section (qualitative data). Of the total sample, 97.8% of the students expressed a high overall satisfaction with simulated video consultations, highlighting their practical utility and positive learning outcomes. From the students’ comments, two main themes and their related categories emerged: advantages (satisfaction and enjoyment, learning, and calmness during simulated scenarios), and disadvantages (technical issues and technical skills development). Simulated video consultations may be considered as one more high-fidelity simulation teaching option. Nursing students should be trained in this modality of healthcare to face the challenge brought on by its increased use in healthcare services, beyond the specific adaptation of clinical simulation sessions due to the closure of universities during this pandemic.

## 1. Introduction

The pandemic experienced worldwide due to the novel coronavirus disease 2019 (COVID-19) has created great concerns for health services [1,2] and caused the implementation of public health measures to reduce the spread of the virus [3,4,5], which included limiting human contacts [2]. Consequently, many governments had to regulate social distancing with measures starting in March 2020 that increasingly restricted teaching practices [6]. In Spain, the government declared the state of alarm on 15 March 2020, using Royal Decree 463/2020 [7] for the management of the healthcare crisis situation owing to the COVID-19 pandemic, by calling for home confinement of the entire population, and including the closure of schools and universities.

To this end, telemedicine systems proliferated, significantly increasing the number of video consultations [8,9]. Among the different telemedicine options, video consultations are being implemented in many countries as a digital health strategy [10,11]. This modality of health care has provided multiple benefits such as avoiding agglomerations due to social distancing restrictions [12], patient satisfaction [13,14,15] and cost reduction [16,17].

Before this pandemic, video consultations were mainly used with patients who had problems accessing the healthcare services [18], for medical consultations in primary and hospital care [19,20], and for chronic disease conditions [21,22,23]. In addition, video consultations were also used for communication between healthcare professionals and clinicians [24]. During this pandemic, video consultations proliferated and their use expanded to other medical conditions and situations [11,13,24,25,26,27], with telemedicine now widely used. In fact, this pandemic has created the need to integrate these telemedicine systems into national health systems [10]. Consequently, training for healthcare professionals is needed [28,29], so they may adequately deal with the possible new challenges of this new modality of health care. In this sense, it should be noted that the interactions in video consultations between patients and healthcare professionals are quite different from an in-person consultation, so these professionals must be prepared to connect and adequately start a video consultation, properly manage possible disruptions during the conversation with the patient, connection failures and latency time during the conversation [24].

Furthermore, this pandemic has become a great challenge to education due to the suspension of face-to-face classroom sessions and closure of education centers [7]. Consequently, these centers have been forced to adapt to new technologies, signifying an opportunity for developing alternatives to achieve the learning objectives planned for each subject. It should be noted that educational institutions have a unique opportunity to bridge this current gap in clinical education with telemedicine, creating interesting proposals for the future [30,31]. More specifically, health sciences students have been particularly affected by this situation due to the suspension of their clinical practices in all healthcare centers and laboratory practices, and the development of high-fidelity clinical simulation sessions, with high-fidelity referring to simulation experiences that are extremely realistic and that provide a high level of interactivity and realism to the learners [32]. This teaching methodology has been demonstrated to be an effective tool for evaluating competencies and clinical performances in both students and healthcare professionals, being an essential part of their training and education [33,34].

In response to this situation, we considered it necessary to adapt our high-fidelity clinical simulation sessions to the new reality in education. Thus, a teaching tool based on high-fidelity simulation, i.e., simulated video consultations, was implemented during the COVID-19 confinement. In addition, these simulated consultations also seemed to be a perfect option for students to practice simulated video consultations for training and for adapting to this booming healthcare modality [35,36]. Thus, during this time, we implemented simulated video consultations utilizing scenarios according to the current reality of this pandemic.

Therefore, the purpose of our study was to explore undergraduate nursing students’ satisfaction and perceptions of simulated video consultations using high-fidelity simulation methodology.

## 2. Materials and Methods

### 2.1. Study Design

We conducted a descriptive cross-sectional study using a mixed-method (both quantitative and qualitative data were assessed) to analyze undergraduate nursing students’ perceptions of simulated nursing video consultations.

### 2.2. Setting and Sample

The study was carried out at a public university in Almeria (Spain) where this simulation modality was implemented, and it included all 3rd-year undergraduate students enrolled in the nursing degree (113 students). The nursing study program at this university is structured into four academic years, and the high-fidelity clinical simulation methodology is implemented in the third and fourth years. Of these, 93 nursing students chose to participate in the study (82.3% response rate).

### 2.3. Simulation Design Process

The implementation of this simulation methodology followed the International Nursing Association for Clinical Simulation and Learning (INACSL) Standards of Best Practice: Simulation^SM^ [37,38,39,40]. In this way, all the stages included in the high-fidelity clinical simulation were accomplished: pre-briefing (establishment of a psychologically-safe learning environment), briefing (previous information related to the simulated scenario), simulated scenario (performance of simulation experience), and debriefing (analysis and discussion of clinical performance during the simulated scenario). These utilized a structured and supported approach, the Gather, Analyze and Summarize (GAS) debriefing tool and the plus-delta technique that allows the observers to differentiate good behaviors (+) from subpar behaviors (Δ) [41]. All of these stages were developed using a virtual platform of online video conferences provided by the university, namely Blackboard Collaborate Launcher^TM^.

A total of six simulated scenarios were designed related to basic healthcare at patients’ homes (all patients were simulated). These simulated scenarios addressed the following clinical cases: a child with febrile syndrome, a bed-ridden patient with a pressure ulcer, a post-surgical patient (laparoscopic cholecystectomy), a child diagnosed with attention deficit hyperactivity disorder (ADHD), a patient diagnosed with arterial hypertension, and a woman with an anxiety disorder (potential case of gender-based violence). All standardized patients (actors and actresses who performed the role of patient according to the Association of Standardized Patient Educators Standards of Best Practice [42]) were confined during the COVID-19 pandemic, so all the specific issues they raised about its adequate management and about protection measures were attended to, aside from the reason behind each consultation. Although the standardized patients changed during the different simulated scenarios, all of them were facilitators in clinical simulation. They were chosen for their experience in this methodology and were trained to play their roles in each simulated scenario, ensuring a high-fidelity level for the simulation experience [42].

Lastly, all nursing students completed three simulation sessions which lasted four hours each (one session of pre-briefing and two sessions where six simulated scenarios were performed, totaling 12 h) and were divided into eight groups (12–16 students). Consequently, each group was divided into six operational work teams (two–three students), who performed a complete simulated scenario together, portraying the role of nursing professionals. While a work team was performing a simulated scenario, the rest of the work teams observed in the debriefing room, learning from the mistakes of their classmates. 

### 2.4. Data Collection

The study was carried out between 2 April and 21 May 2020. On the one hand, the quantitative data were collected by employing the Satisfaction Scale Questionnaire with High-Fidelity Clinical Simulation [43]. Using this questionnaire, we determined the nursing students’ satisfaction with the simulated video consultations. A total of 33 items were included in this questionnaire, which had to be answered using a 5-point Likert response scale (from 1 = strongly disagree to 5 = totally agree). The internal consistency obtained by its creators was satisfactory (Cronbach’s value = 0.920) [43]. On the other hand, the qualitative data were collected from the nursing students’ comments and opinions expressed in an observations section included at the end of the previously-mentioned satisfaction questionnaire. In this section, all the students could express their views on any matter related to the recent completed simulation experience.

### 2.5. Data Analysis

Both quantitative and qualitative data were analyzed. Firstly, the descriptive statistics of the sociodemographic data and each item included in the satisfaction questionnaire were calculated (mean, standard deviation and percentages). This descriptive analysis was performed using IBM SPSS Statistics Version 24.0 software for Windows (IBM Corp., Armonk, NY, USA). Secondly, all qualitative data collected from the students’ comments and opinions were analyzed independently by two researchers. The ATLAS.ti 8 software (Scientific Software Development GmbH, Berlin, Germany) was used for storing, managing, classifying and organizing all the information contained in the qualitative data. Lastly, a thematic analysis was performed for identifying reiterated words, sentences, or ideas in order to be grouped, first into themes and then into categories [44]. In addition, and to ensure anonymity, the participants were numerically labeled in chronological order according to the date of the interview, preceded by the letter “S” (student).

### 2.6. Ethical Considerations

This study was carried out following the ethical principles for medical research of the international Declaration of Helsinki [45] and the data protection policy included in current Spanish legislation [46]. In addition, this study was approved by the Research and Ethics Board of the Department of Nursing, Physiotherapy, and Medicine at the university (nº EFM 75/2020). All nursing students were informed about the study and those who accepted to participate voluntarily signed a written consent. 

## 3. Results

In our study, 93 undergraduate nursing students aged between 20 and 44 years old (mean = 22.14; SD = 6.568) participated, and most of them were women (*n* = 76; 81.7%). Table 1 shows the descriptive data and frequencies obtained in the analysis of each item contained in the satisfaction questionnaire utilized. It should be noted that the five response options were grouped into three scales, as their results were quite similar, facilitating analysis (‘strongly disagree’/‘in disagreement’, ‘indifferent’, and ‘in agreement’/‘completely agree’). In this way, nursing students mainly scored most highly items within the ‘in agreement’/‘completely agree’ range (scores higher than 90%). In this sense, students provided higher scores in the items ‘practical utility’ (100%), ‘I have learned from the mistakes I made during the simulation’ (98.9%), the three items related to the debriefing phase (98.9%), and ‘overall satisfaction with the sessions’ (97.8%). By contrast, they provided low scores for the items ‘I became upset during some of the cases’ (19.4%), ‘I have improved my technical skills’ (49.9%), and ‘simulation has made me more aware/worried about clinical practice’ (63.43%). Regarding the internal consistency of the questionnaire, we obtained a Cronbach’s value quite similar to that obtained by its creators (Cronbach’s value = 0.922), indicating a satisfactory reliability.

Regarding the students’ observations collected in the questionnaire, all the nursing students contributed with short comments. Two analysis themes were identified: advantages and disadvantages of this simulation modality and their corresponding categories. Table 2 shows all categories identified after thematic analysis, including examples of significant quotes provided by the participants.

### 3.1. Theme 1. Advantages of Simulated Video Consultations

Nursing students described multiple advantages after experiencing simulated video consultations. Specifically, the following four categories were identified (ordered according to frequency of mention):

**Category** **1.1.** *Satisfaction and enjoyment: The participants expressed high satisfaction and enjoyed the implementation of this simulation modality. It was positively valued as an alternative to face-to-face high-fidelity clinical simulation sessions by nursing students. In addition, they considered this simulation modality not only as a good adaptation to the situation caused by the COVID-19 pandemic., but also a good method for practicing a modality of health care that may be needed in their future professional career.*

**Category** **1.2.** *Learning: The nursing students ascribed value to the learning acquired through simulated video consultations, considering that this modality may be used during their future clinical practice. They also considered that this modality contributed to increasing and/or reinforcing their learning of non-technical skills (communication, active listening, appearance, empathy, and teamwork), promoting health education, as all the technical skills required during the simulated scenarios had to be explained to the standardized patient to mitigate the inability to perform them in a face-to-face clinical simulation session. However, they also described traditional educational aspects which may be acquired in a typical clinical simulation session, such as practicing in a realistic environment and learning from errors.*

**Category** **1.3.** *Calmness during simulated scenarios performance: Participants indicated that performing simulated scenarios at home using a computer may have contributed to generating less nervousness.*

### 3.2. Theme 2. Disadvantages of Simulated Video Consultations

Participants focused their comments about the disadvantages of this simulation modality on two key points, which were identified as the following two categories (ordered by frequency of mention):

**Category** **2.1.** *Technical issues: Nursing students indicated the Internet connection as a disadvantage, as video consultations require technological resources that must function properly to provide adequate health care.*

**Category** **2.2.** *Technical skills development: They highlighted the inability to perform clinical techniques required in simulated scenarios owing to its virtual format.*

## 4. Discussion

When this simulation methodology was carried out, there was a shortage of research studies that had implemented high-fidelity simulation in video consultations, perhaps because it was not considered necessary until the present. However, the COVID-19 pandemic has changed the reality of healthcare services and video consultations are currently considered as the future of healthcare, although they have also become the immediate present [28,47]. Therefore, we consider that the training and educating of both nursing students and nursing professionals in this modality of healthcare as being essential to adapt them to new healthcare demands, beyond the specific adaptation of clinical simulation sessions for students due to the closure of universities during this pandemic.

Our results indicate a high satisfaction with simulated video consultations (97.8%) supported by the students’ comments, which highlighted their great satisfaction with and enjoyment of the practice and its adaptation of the format for performing clinical simulation sessions during the suspension of in-classroom activities. Another advantage expressed was related to the opportunity to learn from errors and practice in a realistic environment during the simulated scenarios. All of these results are congruent with other studies that obtained a high-level of satisfaction and positive learning outcomes from learners using clinical simulation methodology in face-to face sessions [43,48,49,50]. Particularly, nursing students recognized the important and relevant role of the debriefing phase in their learning, with this result being consistent with other studies [51,52].

Furthermore, the nursing students ascribed value to the learning acquired through simulated video consultations, considering that this modality could be used in their future clinical practice. In addition, our quantitative data showed that all students highlighted the practical utility of this simulation experience (100%). In this sense, it should be noted that this pandemic has challenged health systems worldwide, increasing the use of telemedicine services and in particular the wide use of video consultations [53]. In this way, training in this modality of health care is necessary to adequately manage a video consultation and provide high-quality health care [28]. Our students also considered that this modality contributed to increasing and/or reinforcing their learning of non-technical skills (communication, active listening, appearance, empathy, and teamwork). Clinical simulation also helps with developing these skills, although more research is recommended in this field to assess the development of non-technical skills through virtual simulation modalities [54]. However, since this modality requires healthcare professionals to be more responsive and cautious in order to achieve results similar to face-to-face consultations, the learning of non-technical skills may be increased [55,56].

In previous studies related to face-to-face clinical simulation sessions, learners often expressed high levels of anxiety [57,58]. However, our students describe being calm during the simulated scenarios (68.8%). Thus, our quantitative results were opposite from previous studies, although our qualitative results reinforced our finding, indicating that conducting simulated scenarios at home helped students to be in a safer environment. In this sense, to create a safe environment during simulated scenarios in our study, current recommendations and standards defined by the literature were followed [37,59,60].

However, our students perceived both technical issues and technical skills development as disadvantages of this methodology. Although the use of clinical simulation methodology has been demonstrated to help and improve clinical skills development [33,34], our results showed that simulated video consultations were not adequate for this. However, these disadvantages were quite similar to those indicated by healthcare professionals in real video consultations, who also complained about the inability to perform physical exams and clinical techniques or procedures. In this sense, it should be noted that new platforms and devices are currently being developed to adapt some clinical procedures [26,56]. In addition, this modality of healthcare requires some technological resources, so technological difficulties are the most worrying problem among healthcare professionals who hold video consultations in clinical practice [17,28,56,61]. In particular, these technical problems were aggravated for our students owing to the overloading of the internet connection during the confinement.

The main limitations of our study are related to technical problems during simulated video consultations, and these problems were aggravated by the internet connection overload during the COVID-19 confinement, as both tele-working and online classrooms were widespread. However, technical problems usually occur also in real-life video consultations, and their effectiveness is related to adequate network access and the correct functioning of technology [21,22,56]. Lastly, the high student satisfaction with simulated video consultations obtained in our study should be confirmed by other studies, so more research is needed in this field. In this sense, future studies should also analyze the instructors’ satisfaction with this methodology, assess the acquisition of nursing competencies, and, lastly, be expanded to other settings and education centers.

## 5. Conclusions

Clinical simulation based on simulated video consultations was a satisfactory experience for the nursing students who participated in these simulation sessions. They highlighted that it is necessary to train in this modality of healthcare and to learn non-technical skills (such as active listening, communication, empathy and teamwork) to adequately manage a video consultation. In this way, we propose simulated video consultations as one more high-fidelity simulation option and recommend training and educating nursing students in this modality of healthcare to face the challenges brought on by its increased use in healthcare services.

## Figures and Tables

**Table 1:** Descriptive data and frequencies obtained in each item included in the satisfaction questionnaire (*n* = 93).

Item	Mean (SD)	Strongly Disagree/In Disagreement	Indifferent	In Agreement/Totally Agree
1. Facilities and equipment were real	4.01 (0.993)	6.5%	16.1%	77.4%
2. Objectives were clear cases	4.58 (0.538)	0%	2.2%	97.8%
3. Cases recreated real situations	4.80 (0.456)	0%	2.2%	97.8%
4. Timing for each simulation case was adequate	4.09 (0.868)	6.5%	14%	79.5%
5. The degree of cases difficulty was appropriate to my knowledge.	4.24 (0.728)	3.3%	4.3%	92.4%
6. I felt comfortable and respected during the sessions	4.65 (0.654)	2.2%	3.2%	94.6%
7. Clinical simulation is useful to assess a patient’s clinical simulation	4.41 (0.679)	1.1%	7.5%	91.4%
8. Simulation practices help you learn to avoid mistakes	4.56 (0.580)	1.1%	1.1%	97.8%
9. Simulation has helped me to set priorities for action	4.37 (0.672)	1.1%	7.5%	91.4%
10. Simulation has improved my ability to provide care to my patients	4.25 (0.670)	1.1%	9.7%	89.2%
11. Simulation has made me think about my next clinical practice	4.63 (0.547)	0%	3.2%	96.8%
12. Simulation improves communication and teamwork	4.55 (0.617)	1.1%	3.2%	95.7%
13. Simulation has made me more aware/worried about clinical practice	3.54 (1.113)	20.5%	16.1%	63.4%
14. Simulation is beneficial to relate theory to practice	4.52 (0.583)	0%	4.3%	95.7%
15. Simulation allows us to plan the patient care effectively	4.34 (0.651)	1.1%	6.5%	92.4%
16. I have improved my technical skills	3.04 (0.887)	33.7%	17.2%	49.9%
17. I have reinforced my critical thinking and decision-making	4.37 (0.527)	0%	2.2%	97.8%
18. Simulation helped me assess patient’s condition	4.39 (0.532)	0%	2.2%	97.8%
19. This experience has helped me prioritize care	4.32 (0.611)	1.1%	4.3%	94.6%
20. Simulation promotes self-confidence	4.42 (0.614)	1.1%	3.2%	95.7%
21. I have improved communication with the team	4.40 (0.630)	1.1%	4.3%	94.6%
22. I have improved communication with the family	3.94 (0.832)	3.3%	21.5%	75.2%
23. I have improved communication with the patient	4.44 (0.561)	0%	3.2%	96.8%
24. This type of practice has increased my assertiveness	4.28 (0.682)	1.1%	9.7%	89,3%
25. I became nervous during some of the cases	2.32 (1.199)	68,8%	11.8%	19.4%
26. Interaction with simulation has improved my clinical competence	4.25 (0.583)	0%	7.5%	92.5%
27. The teacher gave constructive feedback after each session	4.76 (0.498)	1.1%	0%	98.9%
28. Debriefing has helped me reflect on the cases	4.77 (0.492)	1.1%	0%	98.9%
29. Debriefing at the end of the session has helped me correct mistakes	4.74 (0.464)	0%	1.1%	98.9%
30. I knew the cases’ theoretical side	4.56 (0.616)	1.1%	3.2%	95.7%
31. I have learned from the mistakes I made during the simulation	4.69 (0.489)	0%	1.1%	98.9%
32. Practical utility	4.62 (0.509)	0%	0%	100%
33. Overall satisfaction with the sessions	4.62 (0.624)	1.1%	1.1%	97.8%

**Table 2:** Themes and categories identified after thematic analysis, including examples of significant quotes provided by participants.

Themes	Categories	Student Discourses (Student = S)
Theme 1. Advantages of simulated video consultations	Category 1.1. Satisfaction and enjoyment	*“I liked it even more than I thought I would have, and I have learned and enjoyed it a lot”* (S33). *“I had fun seeing how my peers acted, thinking about what I would have done and what should be corrected, so my experience has been satisfactory, and I’ve learned not only the theory to address, but also attitudes and how to manage a situation that I may not have experienced before: I have gained resources and knowledge”* (S46) *“A complete novelty, of which I’m grateful to have been a part of, as they had never spoken to us about this possibility as an interview method and to be in contact with patients, so that I am grateful in light of the future”* (S83) *“It has been a good alternative in light of the current situation we are facing”* (S88)
	Category 1.2. Learning	*“Realism, correcting mistakes, empathy, improvement of attitudes and skills, trust, not being embarrassed anymore, non-verbal language techniques. Promoting group participation”* (S10) *“Learn how telehealth will be, as it is something that is being utilized in the health centers due to the current situation we are in, and I think it is important to know how to create a good environment for the patient in distance health services, because sometimes it is complicated”* (S11) *“I have learned new communication skills, to pay attention to the verbal and non-verbal language, to listen to their worries and solve doubts, to explain myself using an easy and clear language, to create comfortable surroundings, and most of all to relax myself and deal directly with the patient. This has allowed me to have an idea about how to manage possible situations that I may experience with real future patients”* (S49) *“It is an approach to a real situation with many realistic issues, it provides options for developing communication and psychological techniques, it lets us learn from our mistakes before making them in real life, and it makes you become truly involved with the patient, searching for all the means to help him or her”* (S50).
	Category 1.3. Calmness during simulated scenarios performance	*“You feel less nervous when you are performing the simulation from your house and within your comfort zone”* (S52) *“I think we have felt less embarrassed and with more confidence when performing the simulation through the screen”* (S65) *“When being in my own room, it didn’t feel like an exam, and this has resulted in me being more calm”* (S71). *“Less nervousness”* (S74)
Theme 2. Disadvantages of simulated video consultations	Category 2.1. Technical issues	*“Maybe the main problem was the internet connection, but this is not dependent on the simulation”* (S21) *“Maybe the quality of the connection, but there isn’t much to improve”*. (S30) *“The connection problems (could be a positive issue if you know how to deal with it”* (S57)
	Category 2.2. Technical skills development	*“Due to the current situation of having to do the simulation online, practical skills have not been able to be demonstrated”*. (S7) *“We have not been able to improve the technical skill in the same way”* (S9) *“It’s difficult to learn practical skills with an online simulation, but many other things are learned”*. (S60)
